# Neuroprotective Effects of *Rosa damascena* Extract against Aluminum Chloride-Induced Brain Damage in Rat Offspring

**DOI:** 10.1155/2023/5342849

**Published:** 2023-12-04

**Authors:** Leila Beigom Hejazian, Seyed Mohammad Hosseini, Alireza Salehi

**Affiliations:** ^1^Cellular and Molecular Biology Research Center (CMBRC), Babol University of Medical Sciences, Babol, Iran; ^2^Department of Human Anatomy, Faculty of Medicine, Babol University of Medical Sciences, Babol, Iran; ^3^Department of Pathology, Babol Branch, Islamic Azad University, Babol, Iran

## Abstract

Aluminum (Al) is a popular metal in the industry, and its usage has greatly increased recently. The dose of this metal has been proven to be toxic to rats, but its effects on the offspring of the original receivers and prevention methods to reduce this damage are unknown. *Rosa damascena* is a well-known plant for its high antioxidant capabilities. In this study, the protective effect of *Rosa damascena* extract (RDA) on aluminum-induced lesions in the brain tissue of a rat offspring was investigated. In this regard, female rats were divided into seven groups, including the control group, the sham group, the aluminum group at the dose of 100 mg/kg, the extract groups at the doses of 500 and 1000 mg/kg, and the treatment groups that received the extract and Al at the same doses. After the treatment ended, the offsprings were subjected to exploratory behavioral tests, and finally, the tissues of the brain including the cerebral cortex, hippocampus, and hypothalamus were pathologically examined. It was observed that RDA at the dose of 1000 mg/kg reduced the malondialdehyde (MDA) and acetylcholinesterase (AChE) levels significantly (*P* < 0.0001), while raising the catalase and FRAP indices in Al-treated rats. Moreover, it increased neuronal counts significantly and reduced necrosis and vacuolar degeneration in both the cortex and hippocampus compared to the Al-receiving group. In addition, the administration of RDA 1000 improved the behavioral test scores of the offspring. In conclusion, RDA can effectively reduce Al-induced damage in the brain tissue of the offspring.

## 1. Introduction

Aluminum is the third most commonly used metal in the industry, and it enters the human body through various foods and products [1]. Al can act as a neurotoxin that has a wide effect on multiple organs [2]. It has been observed that aluminum easily enters the brain and accumulates in it, and the highest amount is seen in younger rats, indicating the importance of accumulating this metal in the early life of the animal [3]. Some of the diseases that have been proven to be associated with aluminum are Parkinson's disease, dementia, and Alzheimer's [4–7]. This metal can also cause oxidative stress by damaging the lipid membrane, proteins, and enzymes of the antioxidant defense system [8]. In a previous study, it was observed that injection or oral administration of this metal can cause lesions in pregnant rats and their infants [2]. An earlier study showed that Al can induce histopathological damage, oxidative stress, and behavioral dysfunction in rats [9].

Despite being a natural procedure, pregnancy is one of the sources of stress in females [10, 11], and aluminum toxicity aggravates this stress. It has been observed that aluminum not only passes through the blood-brain barrier but also increases its permeability and the destructive effects of other harmful chemicals [12]. Furthermore, it has been observed that aluminum can cause serious damage to the fetus during pregnancy [13]. The effects of aluminum on the brains of fetuses can cause different lesions, which can be seen clinically in pathological examinations and behavioral tests [2]. It has been observed that this metal causes oxidative stress in various tissues [1].

The consumption of natural anti-inflammatory, anticancer, and antioxidant agents has become very popular in current medicine [14]. RDA is known as a rich source of antioxidants [15, 16]. This extract contains many phenolic substances that scavenge and absorb tissue free radicals and significantly reduce the amount of tissue reactive oxygen species (ROS) [17]. ROS resulting from normal aerobic metabolism are potentially harmful; therefore, these free radicals are usually removed or disabled by antioxidant groups *in vivo.* Other components of this extract, such as terpenes, glycosides, flavonoids, and anthocyanins, have shown a great promise in medicine [18].

It has been previously observed that RDA can significantly protect rats against Al-induced oxidative stress [9]. However, this study aimed to investigate the potential of RDA in Al-induced vertically transmitted lesions in rat offspring. Accordingly, we used the same protocol to induce Al-induced lesions in female rats but evaluated the offspring pathologically.

## 2. Materials and Methods

### 2.1. Animals and Ethics

This study aimed to evaluate the protective potential of RDA for aluminum-induced brain damage in rat offspring. Female rats were mated with healthy male rats, and the offspring were randomly divided into seven groups of 10. After obtaining the required infants, they were kept at 20°C with 12 hours of dark/light cycle and 55–65% humidity [19]. Moreover, in addition to the ARRIVE guidelines [20], the Ethics Committee of Babol University of Medical Sciences approved the experimental methods (approval ID Mubabol. rec. 1394.24).

### 2.2. Aluminum and RDA Preparation

Aluminum chloride was purchased from Sigma-Aldrich (St. Louis, MO, USA). *Rosa damascena* used in this experiment was purchased from the city of Kashan, central Iran. To prepare the plant extract, 2 liters of pure ethanol was added to 100 g of dried flower powder. The mixture was then placed in the laboratory at 25°C. After 48 h, the mixture was filtered and the solvent was evaporated by using a rotary evaporator at 50°C [17]. Then, the extract was analyzed by using a gas chromatography (GC)-mass spectrophotometer (MS) system (Agilent Technologies 7890A) to identify its natural compounds [21].

### 2.3. Study Design

**Figure 1:**
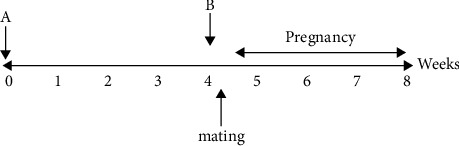
Timeline of the study. A: beginning of RDA administration; B: beginning of the administration of aluminum.

Female rats were purchased from the Pasteur Institute and randomly divided into seven groups of 10. In the first 4 weeks, RDA was administered to groups 3, 4, 6, and 7. Then, in the 5^th^ week, Al was administered to groups 5, 6, and 7. Male rats were employed for mating one day after the administration of Al. So, the mating of all rats had the same starting date. Pregnant rats were then treated until delivery. Approximately eight weeks after the beginning of the project, the offspring were delivered and evaluated (Figure 1). The following are the details of the groups:

  Group 1 (control): offspring of females that did not receive any treatment
  Group 2 (sham): offspring of females that received normal saline
  Group 3 (extract 1): the offspring of females that received RDA at a dose of 500 mg/kg [17] for eight weeks
  Group 4 (extract 2): the offspring of females that received RDA at a dose of 1000 mg/kg [17] for eight weeks
  Group 5 (AL): offspring of females that received aluminum for four weeks at a dose of 100 mg/kg before mating [22]
  Group 6 (treated 1): offspring of females that received both RDA at a dose of 500 mg/kg and aluminum at a dose of 100 mg/kg at the same time point
  Group 7 (treated 2): offspring of females that received both RDA at a dose of 1000 mg/kg and aluminum at a dose of 100 mg/kg at the same time point

### 2.4. Shuttle Avoidance

A shuttle avoidance box was used to assess the learning and memory of the offspring. The shuttle box was placed in a dimly lit position. The metal grid floors of both compartments were weight sensitive [23].

### 2.5. Y-Maze Test

All test attempts were performed between 3 p.m. and 6 p.m. The Y-maze was a Y-shaped plexiglass, which holds a cage with a 40 cm length, 30 cm height, and 15 cm width. Rats were allowed to freely discover the maze for an 8 min observation period. Air circulation equipment in continuous operation provided masking noise of 40 dB. Alternation was defined as successive entries into the three arms of overlapping triplet sets. The alternation percentage was calculated as the ratio of actual to possible alternations (defined as the total number of arm entries minus two). First, each rat was placed in the start arm and then released to choose one of the other arms. Then, the rat was trapped within the selected arm for 30 s, placed back in the start arm for another 30 s, and then released to choose one of the other arms again. If a different arm was selected by the rat, it was scored as alternating [24, 25].

### 2.6. Oxidative Stress Marker Estimation

The tissues were prepared by using an approved method from a previous study, and the stress markers were examined separately using special kits [9].

#### 2.6.1. AChE

A kinetic photometric method was used for the AChE activity assay. Cholinesterase can break down butyrylthiocholine to thiocholine, which reacts with 5,5′-dithiobis-2-nitrobenzoate, forming the yellow product of 5-thio-2-nitrobenzoate with an absorbance at 412 nm. The rate of change in the absorbance was regulated to 1 g of tissue. Cholinesterase activity was calculated by using a molar extinction coefficient value of 13.6 mM expressed as U/mg protein. A human blood serum sample was used as a positive control. Cholinesterase activity was calculated using the extinction coefficient of the thiolate dianion of DTNB at 412 nm, which is produced by the enzymatic hydrolysis of acetylthiocholine iodide [9].

#### 2.6.2. FRAP

The FRAP value was measured by comparing the change in absorbance at 593 nm in a test reaction tube containing the mixtures that included tissue samples with 2,4,6-tri-(2-pyridyl)-s-triazine with a defined ferrous ion concentration. When free radicals were reduced to the ferrous form (Feп) at lower pH values, the FeШ-2,4,6-tri-(2-pyridyl)-s-triazine complex produced an intense blue color product with absorption at 593 nm. Practically, conditions that are favorable for complex development are provided in the presence of reductants 0 (antioxidants), which allow color to develop. A standard solution of ferrous sulfate (Feп100 to 2000 mM) was prepared in distilled water. The FRAP value was expressed as mmol tissue weight [9].

#### 2.6.3. MDA

Tris-HCl buffer was applied for diluting the tissue homogenate (0.5 ml); then, it was incubated at 37°C for 2 hours. 1 ml of cold trichloroacetic acid (TCA) was added, vortexed, and centrifuged at 800 × *g* for 10 min. Then, up to 1 ml of the supernatant was added. Thiobarbituric acid (TBA; 1 mL) and the reaction mixture were placed in a boiling water bath for 15 min. A pink-colored complex was formed, and its absorbance was read at 532 nm. An extinction coefficient of 1.56 × 105 M^−1^·cm^−1^ for the MDA-TBA chromophore was used to calculate the amount of MDA formed (index of lipid peroxidation), and the results were expressed as nmol/mg protein [9].

#### 2.6.4. Catalase

Catalase activity was measured based on its ability to decompose H_2_O_2_ in brain tissue [26]. H_2_O_2_ decomposition was assessed by measuring the reduction in absorbance at 240 nm. Thus, hydrogen peroxide was used at a final concentration of 19 mM. In addition, 50 mM of phosphate buffer (pH 7) was used as a substrate and an alternative substrate in the blank solution was employed consecutively. The reaction was initiated by adding H_2_O_2_, and the decrease in the absorbance was evaluated by using a spectrophotometer (Pharmacia, Novaspec II, and Biochrom, England) at 240 nm for 30 seconds. The values were expressed as U/mg protein, according to a previously described method [9].

#### 2.6.5. GSH

Glutathione (GSH) estimation was performed by employing the Ellman method. For this purpose, 1 ml of the supernatant was collected after sedimentation of the brain homogenate (0.5 ml of brain homogenate with 2 ml of 5% TCA). Then, 0.5 ml of Ellman's reagent (0.0198% DTNB in 1% sodium citrate) and 3 ml of phosphate buffer (1 M, pH 8.0) were added. The developed color was observed at 412 nm. A standard curve was drawn by using known levels of reduced GSH concentration described as mg/g of tissue [9].

### 2.7. Pathological Examination

Different parts of the brain were sampled, fixed in formalin buffer 10%, and processed serially. The sections were then stained with H&E and observed using a CX23 microscope [27]. Neurons were counted by histopathological examination of tissue samples under a microscope histopathologically [28, 29]. In addition to H&E staining, hippocampal tissues were stained with Nissl [30].

### 2.8. Data Analysis

Data are expressed as the mean ± SE. Latency data and covered distance during the training days were analyzed using repeated measures within groups and two-way analysis of variance (ANOVA), followed by a Tukey post hoc test to compare the groups [31]. In addition, Kruskal–Wallis and Mann–Whitney *U* tests were used for histopathological scoring differences between the groups. Statistical comparisons were performed using the SPSS 27 version [21]. A *P* value of less than 0.05 was determined as a significant difference [32].

## 3. Results

### 3.1. GC-MS of RDA

The extract was analyzed, and the most prominent compounds with the highest proportion were furfural (28.14%), quinic acid (22.90%), geraniol (9.39%), citronellol (6.35%), phenethyl alcohol (5.48%), clionasterol (3.59%), nonadecane (2.98%), ethanone (2.42%), heneicosane (1.78%), propylamine (1.55%), furfuryl alcohol (1.12%), and octadiene (1.12%).

### 3.2. Behavioral Tests

**Figure 2:**
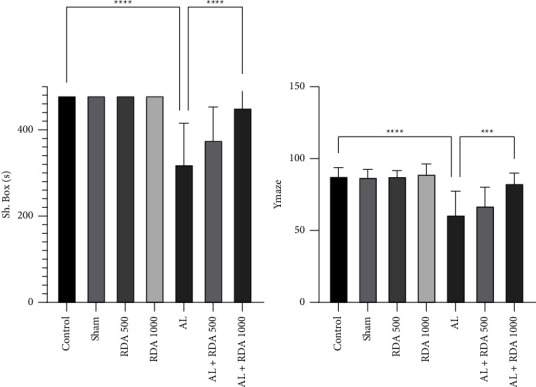
Comparison between behavioral tests in all groups. ^*∗∗∗*^*P* < 0.001 and ^*∗∗∗∗*^*P* < 0.0001: significant compared to the AL group. ^*∗*^*P* < 0.05: significant difference between treatments. All results are expressed as the mean ± standard deviation. N = 10.

It can be clearly seen that offspring whose mothers were treated via Al had a significantly (*P* < 0.0001) lower successful attempt to discover the maze than those in the control group, and it was harder for them to remember the path. However, the AL + RDA 1000-treated group was significantly (*P* < 0.0001) more successful than the AL group. Moreover, the Al-treated offspring spent more time in the wrong section of the shuttle box compared to the control group, while the AL + RDA 1000 group spent significantly (*P* < 0.001) more time in the right section, and got less punished via the electric shock (Figure 2).

### 3.3. AChE

**Figure 3:**
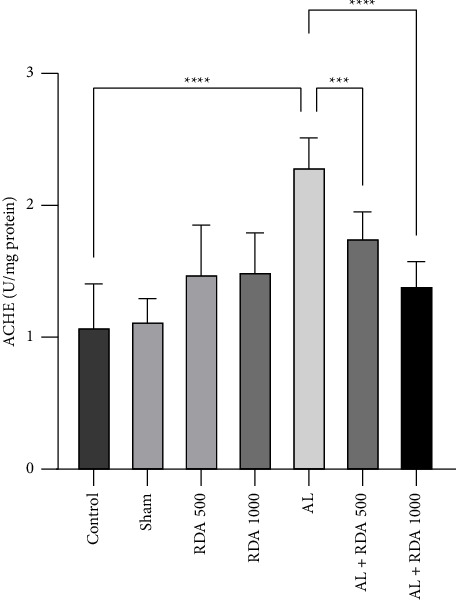
Comparison between oxidative stress marker levels in all groups. ^*∗∗∗*^*P* < 0.001, and ^*∗∗∗∗*^*P* < 0.0001: significant compared to the AL group. All results are expressed as the mean ± standard deviation. N = 10.

As can be seen from the figure 1, AChE was significantly (*P* < 0.0001) higher in the AL group than in the control group. In addition, there was a substantial decrease in the AL + RDA 1000 group compared to that in the AL group (Figure 3).

### 3.4. Oxidative Stress Markers

**Figure 4:**
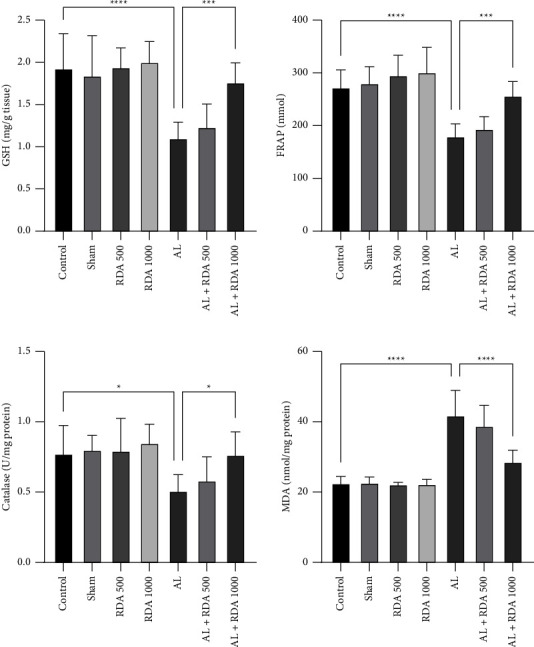
Comparison between oxidative stress marker levels in all groups. ^*∗*^*P* < 0.05, ^*∗∗∗*^*P* < 0.001, and ^*∗∗∗∗*^*P* < 0.0001: significant compared to the AL group. All results are expressed as the mean ± standard deviation. N = 10.

Regarding stress markers, the FRAP value in the AL group dipped significantly (*P* < 0.0001) compared to the control group, which rose in the AL + RDA 1000 group dramatically (*P* < 0.001). The MDA level was also reduced after Al administration, but it went up in the AL + RDA 1000 group. The catalase estimation revealed that the Al can substantially decrease it (*P* < 0.05), while the RDA 1000 showed a significant (*P* < 0.05) rise compared to the AL group. Finally, the GSH level was significantly (*P* < 0.0001) reduced in the AL group compared to the control group, whereas it was increased in the AL + RDA 1000 group positively (Figure 4).

### 3.5. Pathological Examination

**Figure 5:**
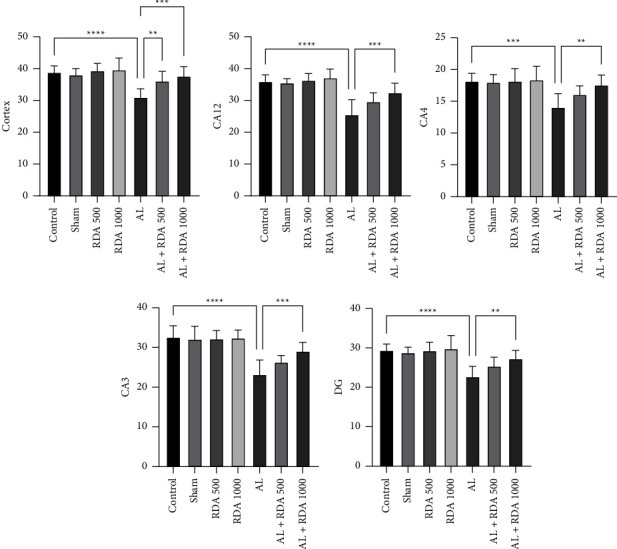
Neuronal counts in different areas of the brain. ^*∗∗*^*P* < 0.01, ^*∗∗∗*^*P* < 0.001, and ^*∗∗∗∗*^*P* < 0.0001: significant compared to the AL group. All results are expressed as the mean ± standard deviation. N = 10.

As shown, the number of neuronal counts in the cortex of the AL group was decreased significantly (*P* < 0.0001) compared to the control group. Following that, in all regions of the hippocampus of the brain, the same trend occurred, and they were all significant. However, in the AL + RDA 1000 group, these numbers were significantly (*P* < 0.001) higher, and it was observed that RDA 1000-treated rats had a better performance than those of the AL group (Figure 5).

**Figure 6:**
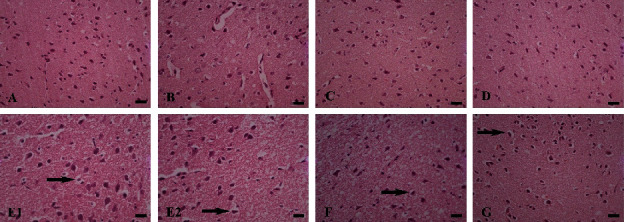
Cortex tissue. Control group (A), Sham group (B), RDA 500 group (C), and RDA 1000 group (D): normal brain tissue. AL group (E1, E2), AL + RDA 500 group (F), and AL + RDA 1000 group (G): vacuolar degeneration (right arrow). H&E staining, ×40 magnifications; scale bar = 100 *µ*m.

**Figure 7:**
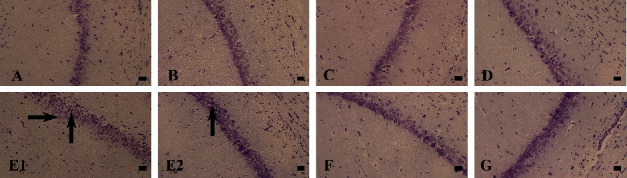
Hippocampus tissue. Control group (A), Sham group (B), E1 group (C), and E2 group (D): normal brain tissue; AL group (E1, E2), AL + RDA 500 group (F), and AL + RDA 1000 group (G): vacuolar degeneration (right arrow), necrosis (up arrow). Nissl staining, ×40 magnifications; bar = 50 *µ*m.

**Figure 8:**
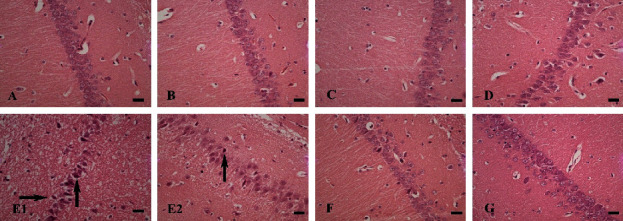
Hippocampus tissue. Control group (A), Sham group (B), E1 group (C), and E2 group (D): normal brain tissue; AL group (E1, E2), AL + RDA 500 group (F), and AL + RDA 1000 group (G): vacuolar degeneration (right arrow), necrosis (up arrow). H&E staining, ×40 magnifications; bar = 100 *µ*m.

In the histological examination, it was observed that in the cortex of the AL group, dramatical necrosis, and vacuolar degeneration occurred compared to the control group (Figures 6–8). In addition, hippocampus examination demonstrated a significant rise in the necrosis and vacuolar degeneration indices compared to the control group. However, in both parts of the brain, substantially fewer lesions were detected in the AL + RDA 1000 group compared to the AL group (Table 1).

## 4. Discussion

In this study, the protective effect of RDA against aluminum-induced brain damage in rat offspring was evaluated, and it was observed that there are some critical benefits against Al with the administration of RDA. As it was observed in our previous study, Al can induce heavy neurotoxicity and oxidative damage in brain cells, while administrating RDA can effectively reduce those lesions. This study focused on the offspring of rats, which were treated with Al, to investigate the amount of neurotoxicity that can transfer from a mother to her offspring. It was previously observed that prenatal exposure to Al can lead to some cell damage [33] and Al accumulation in different tissues [34]. In addition, administrating Al to female rats caused heavy oxidative damage and manipulated behavioral reactions [9].

RDA consists of some powerful natural compounds, which shape its characteristics. Furfural was the first compound, which was detected. It is proven that this compound can improve the efficiency of other substances, and with its high antioxidant properties, it can substantially reduce the harmful effects of chemicals and toxins [35]. The second natural compound was quinic acid, which is proven to be a strong antioxidant [36, 37]. Previous studies strongly suggested using natural antioxidants, such as curcumin and quercetin for reducing oxidative stress, cell dysfunction, and improving neurobehavioral impairment [38, 39].

Chlorogenic acids are a group of phenolic compounds that can be found in diets and have antioxidant activity [40], which is beneficial to neurological organs [41]. These compounds were found in *R. damascena* extract. They are antioxidants [42] and can inhibit acetylcholinesterase activity in some parts of the brain, such as the frontal cortex and hippocampus [43]. Multiple antioxidant substances were found in RDA, including quinic acid, geraniol, citronellol, and furfural, which had the highest proportion. Quinic acid demonstrated a prometabolite activity that can lead to the induction of high levels of nicotinamide and tryptophan [44]. The findings of our study showed Al administration can increase AChE activity, while RDA can downregulate its activity. Geraniol and citronellol proved that they have high antioxidant properties [45, 46]. Moreover, phenethyl alcohol was also found in RDA, which is another anticholinesterase agent [45].

Since acetylcholine (AChE) is a critical neurotransmitter in the memory and learning process, the level of AChE was measured in this study. Moreover, low AChE can result in a lower performance and reduced function of the brain in Alzheimer's disease because neurotransmitters are critically required in synapses for the transmission of nerve impulses [47]. In addition, AChE plays an important role in cholinergic transmission. So, cholinesterase may also play a role in Alzheimer's disease [48]. It was observed that the activity of AChE was elevated in the AL group, which was a direct result of aluminum administration [49]. Aluminum is a strong cholinotoxin [50] and can affect acetylcholinesterase levels by accelerating its activity. This effect was accompanied by oxidative stress, which gradually ended in more accumulation of aluminum in the brain, and this could be the reason for increasing AChE and AD clinical signs [51]. However, the combination of multiple natural compounds in RDA led to a significant decrease (*P* < 0.0001) in AChE levels in the RDA1000-treated group compared to the AL group.

Regarding the behavioral tests, it was seen that the offspring whose mothers were treated with Al were suffering from brain damage, and it was harder for them to remember the path or the consequence of choosing the wrong box in the shuttle box. It seemed that Al had caused permanent brain damage by passing through the blood-brain barrier in the offspring [12, 52, 53]. However, RDA at the dose of 1000 mg/kg showed a remarkable potential for improving the scores of the behavioral tests in offspring. It seemed that those whose mothers had received RDA 1000 were less likely to be harmed by the neuronal damage of Al, which could be the result of ROS's protectivity in the brain tissue, which was shown in earlier studies [54, 55]. Since behavioral disorder was also seen in mother rats [9] in addition to the infant in the current study, it can be concluded that Al can be easily transferred from mother to infant through the umbilical cord.

The stress markers were evaluated, and the results suggested that RDA at the dose of 1000 mg/kg can effectively reduce the harmful effects of aluminum at the dose of 100 mg/kg in the second generation. The amount of MDA was significantly higher in the AL group, which demonstrated neuronal differentiation [56] combined with an increased free radical due to lipid peroxidation [57] in the brain tissue of offspring. Moreover, a significant fall in the FRAP value in the AL group was in line with the previously mentioned findings. It is assumed that aluminum has reduced the power of the antioxidant system in the tissue by increasing the ROS and reducing the antioxidant agents such as FRAP [58]. However, in the RDA 1000 group, the oxidant balance was approximately restored, and a substantially higher FRAP value was observed, which is a reliable antioxidant capacity assay [59]. Catalase and GSH were the other estimated markers, which usually decreased during brain damage [60]. Both of these markers had substantially fallen in the AL group, while in the RDA 1000-treated group, the values were much higher. This would be probably due to the antioxidant properties of the RDA and the natural components of it, which effectively reduced the damage and restored the oxidant balance to the tissue.

Histological observations revealed an impactfully reduced number of neuronal counts in the cortex and hippocampus of the AL group offspring, which justified the absence of the normal ability of the offspring to remember or to decide in the employed behavioral tests. However, the number of neuronal counts was higher in the RDA 1000 group than in the AL group, which demonstrated the reason for the better response to the external stimuli in that group. Moreover, this is in line with other findings and it correlated with the higher amount of antioxidant capacity and lower amount of tissue damage in that group. This finding may suggest the high potential of RDA at the dose of 1000 mg/kg for this fascinating impact.

The pathological examination demonstrated the expected neuronal damage, which was practically observed in the behavioral tests. It was shown that the number of neuronal counts fell significantly in the AL group compared to the control group; moreover, they were reduced in different parts of the brain rather than a specific area. Thus, it can be said that Al can spread through the brain tissue by moving through the vascular system and causing some negative impactful changes. Moreover, both necrosis and vacuolar degeneration were dramatically increased in the AL group, which again was in line with other findings. However, RDA 1000 was significantly successful in lowering the damage in the mentioned aspects, which is likely due to its high protectivity characteristics by regulating the antioxidant system [61, 62], reducing the ROS [63, 64], and simultaneously increasing the antioxidant capacity of the body [64, 65].

According to previous studies, antioxidants can play a major role in aluminum-derived damage [66, 67]. It seems that RDA has relatively massive amounts of antioxidants, which helped it to successfully upregulate the antioxidant defense system [68]. This regulation led to significantly lower the brain tissue damage and significantly lower oxidative stress. Consequently, it improved the function of the brain, which was clinically observed during the behavioral tests. It should also be noted that antioxidants can greatly improve brain function by cholinergic modulation [69], so using them as a therapeutic agent will be beneficial in different pathways [70–72].

## 5. Conclusion

Overall, it was observed that there is a significant difference between the offspring of females who had received the Al compared to the control group. It proves that Al can be passed from the mother to the offspring and can meaningfully penetrate the offspring from the mother by passing through the placenta; and then, enter the brain tissue by going through the BBB, and finally, cause various neuronal damages including reduced nodes, necrosis, and vacuolar degeneration. The behavioral tests confirm these impactful damages, which lead to a lower functionality in remembering, getting experience, and learning from mistakes. However, the RDA showed a promising potential at the dose of 1000 mg/kg. It effectively reduced the neuronal damage and improved the ability of the offspring to remember and behave more naturally. In addition, the stress markers were lower in the RDA 1000 receiving group, which showed a substantially strong antioxidant potential.

## Figures and Tables

**Table 1:** Histopathological examination of necrosis and vacuolar degeneration in the cortex and hippocampus tissues of all groups.

	Cortex		Hippocampus	
	Necrosis	Vacuolar degeneration	Necrosis	Vacuolar degeneration
Control	0.10 ± 0.10	0.10 ± 0.10	0.10 ± 0.10	0.10 ± 0.10
Sham	0.10 ± 0.10	0.10 ± 0.10	0.10 ± 0.10	0.10 ± 0.10
AL	1.10 ± 0.23^a,c^	0.80 ± 0.20^a,c^	1.10 ± 0.23^a,c^	0.70 ± 0.15^a,b,c^
AL + RDA 500	0.50 ± 0.17	0.30 ± 0.15	0.50 ± 0.17	0.20 ± 0.13
AL + RDA 1000	0.30 ± 0.15	0.20 ± 0.13	0.40 ± 0.16	0.20 ± 0.13
RDA 500	0.10 ± 0.10	0.10 ± 0.10	0.10 ± 0.10	0.10 ± 0.10
RDA 1000	0.11 ± 0.11	0.11 ± 0.11	0.11 ± 0.11	0.11 ± 0.11
*P* value^*∗*^	0.001	0.010	0.001	0.011

Values are expressed as mean ± standard error. ^*∗*^Significances of cortex and hippocampus lesions between groups (*P* < 0.05; Kruskal–Wallis test). ^a^Statistically significant difference between the control and AL-treated groups. ^b^Statistically significant difference between AL and RDA 500-treated groups. ^c^Statistically significant difference between AL and RDA 1000-treated groups. *N* = 10.

## Data Availability

The data used to support the findings of the study are available from the corresponding author upon request.

## Abbreviations

Al: Aluminum
RDA: *Rosa damascena* extract
MDA: Malondialdehyde
AChE: Acetylcholinesterase
ROS: Reactive oxygen species
GSH: Glutathione.
